# A short note on dynamic programming in a band

**DOI:** 10.1186/s12859-018-2228-9

**Published:** 2018-06-15

**Authors:** Jean-François Gibrat

**Affiliations:** MaIAGE, INRA, Université Paris-Saclay, Jouy-en-Josas, 78350 France

**Keywords:** NGS, Read correction, Semi-global alignment

## Abstract

**Background:**

Third generation sequencing technologies generate long reads that exhibit high error rates, in particular for insertions and deletions which are usually the most difficult errors to cope with. The only exact algorithm capable of aligning sequences with insertions and deletions is a dynamic programming algorithm.

**Results:**

In this note, for the sake of efficiency, we consider dynamic programming in a band. We show how to choose the band width in function of the long reads’ error rates, thus obtaining an $O(N^{\frac {3}{2}})$ algorithm in space and time. We also propose a procedure to decide whether this algorithm, when applied to semi-global alignments, provides the optimal score.

**Conclusions:**

We suggest that dynamic programming in a band is well suited to the problem of aligning long reads between themselves and can be used as a core component of methods for obtaining a consensus sequence from the long reads alone.

The function implementing the dynamic programming algorithm in a band is available, as a standalone program, at: https://forgemia.inra.fr/jean-francois.gibrat/BAND_DYN_PROG.git

## Background

High throughput sequencing technologies are progressing at a very fast pace. Recently, third generation sequencing technologies have been launched on the market and are becoming available to the life science community. These novel sequencing technologies are based on single molecule techniques and are characterized by very long reads exhibiting a high error rate. At the time of writing, Pacific Biosciences (http://www.pacb.com) machines produce reads whose length distribution has a mean of 15 kbp. The longest sequenced reads have a length of about 55 kbp. These reads have an error rate of 10-15% (typically, 3% mismatches, 7% insertions and 4% deletions). These figures are likely to change with the techniques’ improvements, but give a general idea of the characteristics of 3rd generation technologies so far.

These characteristics have a strong impact on the algorithms used to assemble or map the reads produced by these technologies. Current algorithms have been designed to cope with 2nd generation sequencing data, i.e., a very large number of short reads (at most a few hundred-base pair-long) with very low error rates (< 1%). The length of 3rd generation reads and their high error rates, particularly for insertions and deletions, is likely to make these algorithms ill-adapted to efficiently process 3rd generation sequencing technology data. Algorithms better able to cope with reads exhibiting large numbers of insertions and deletions are needed.

## Method

Until now, the only exact algorithm for aligning sequences with insertions and deletions remains the dynamic programming (DP) algorithm proposed by Needleman & Wunsch almost 50 years ago [8]. This algorithm has been modified by Smith & Waterman [9] to provide local alignments in addition to the original global and semi-global alignments. This canonical algorithm is *O*(*NM*) in time and space, where *N* and *M* are the lengths of the two sequences to be aligned. To illustrate this point, if one wants to compare two long reads of length 50 kbp as described above, this requires allocating enough memory for a matrix of 2.5 10^9^ elements. If this is a matrix of floats or integers (4 bytes), this requires 10 GB of memory.

A number of works have been carried out to try alleviating this problem. Following a proposal of Hirschberg [3], different authors have presented algorithms to reduce the space requirement of the algorithm to *O*(*N*) [4, 7]. The time requirement, though, remains *O*(*NM*).

Other groups have addressed the time requirement issue. Masek & Paterson [6] have proposed an algorithm displaying an $O\left (\frac {NM}{\log {N}}\right)$ runtime, but this algorithm was based on a particular scoring scheme consisting of rational numbers only and did not allow local alignments. Crochemore & Rytter [1] proposed a more general-purpose algorithm that overcame these limitations and had an $O(\frac {hNM}{\log {N}})$ time requirement, *h* being the entropy of the sequences. However, so far, there is no known algorithm that achieves the above subquadratic runtime and whose space requirement is linear.

**Fig. 1:**
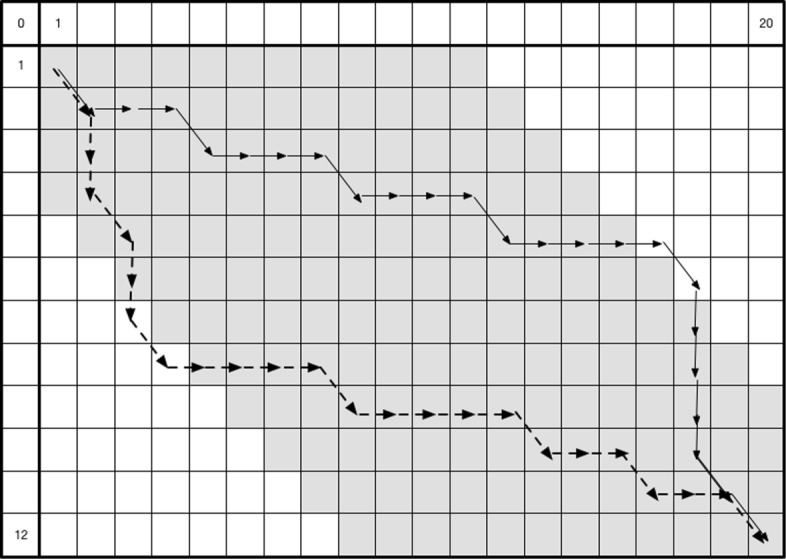
Semi-global alignment in a band. The w-band is shown in gray. The 1st, horizontal sequence has length *l*_1_=20. The second sequence has length *l*_2_=12. The width of the band is *w*=3. This is a *global alignment*, hence the path starts at position [1,1] in the matrix and ends at position [ *l*_2_,*l*_1_]

Fickett [2] noticed that, in the special case where the two sequences to be aligned are highly similar, it is sufficient to perform the dynamic programming algorithm in a band around the main diagonal (see Fig. 1). If the alignment path remains within the band, this algorithm achieves an *O*(*wN*) time and space requirement, where 2*w*+1 is the width of the band and *w*<<*N*. If the alignment path leaves the band, one must restart the algorithm with an increased value of *w*, for instance, by doubling it. This process continues until one is certain that the path remains within the band. In the worst case, the runtime is still *O*(*NM*).

Then two questions arise. How should the band width be chosen? How does one know that this algorithm produces an optimal alignment, i.e., that the alignment path remains within the band?

### Optimal choice of the band width

As shown in Fig. 1, each insertion in the first sequence (or deletion in the second sequence) moves the path one position to the right, conversely a deletion in the first sequence (or insertion in the second sequence) moves the path one position downwards. Given the observed sequencing technology error rates, what is the probability that the path leaves the band of width 2*w*+1 when aligning reads of length *N* due to a random accumulation of steps in a particular direction?

This problem can be modeled as a 1D random walk. Consider a random walk along the *x* axis, starting at position 0. The probability to take one step to the right is *p*_*r*_, the probability to take one step to the left is *p*_*l*_ and the probability to remain stationary is 1−*p*_*r*_−*p*_*l*_. The probability to take *r* steps to the right, *l* steps to the left and to remain stationary *s* times during a walk of length *N* is given by the multinomial distribution:

1 $$ \mathbb{P}(r,l,s) = \frac{N!}{r!\;l!\;s!} \; p_{r}^{r} \;p_{l}^{l} \; (1-p_{r}-p_{l})^{s}  $$

with *r*+*l*+*s*=*N*. To answer the above question, we consider the travelled distance: *d*=*r*−*l* and compute its variance: $\mathbb {V}\text {ar}(d) = \mathbb {E}(d^{2}) - [\mathbb {E}(d)]^{2}$ with:

2 $$ \begin{array}{lcl} {}\mathbb{E}(d) & = & \mathbb{E}(r-l) = \mathbb{E}(r) - \mathbb{E}(l) = Np_{r} - Np_{l}\\ {} \mathbb{E}(d^{2}) & = & \mathbb{E}(\!(r\,-\,l)^{2}) \,=\, \mathbb{E}(r^{2} \,-\, 2rl + \!l^{2}) \,=\, \mathbb{E}(r^{2}) \,-\, 2 \mathbb{E}(rl) \,+\, \mathbb{E}(l^{2}) \end{array}  $$

To evaluate $\mathbb {E}(d^{2})$, we need to compute $\mathbb {E}(r^{2})$, $\mathbb {E}(l^{2})$ and $\mathbb {E}(rl)$.

3 $$ \mathbb{E}(rl) = \mathbb{E}(r)\;\mathbb{E}(l) + \text{Cov}(r,l) = Np_{r}Np_{l} - Np_{r}p_{l}  $$

To compute $\mathbb {E}(r^{2})$ we start with the multinomial distribution moment generating function (MGF):

4 $$ \left[p_{r}e^{t_{r}}+p_{l}e^{t_{l}}+(1-p_{r}-p_{s})e^{t_{s}}\right]^{N}  $$

For the random variable *r*, the second derivative of the MGF is:

5 $$ N(N-1) [\ldots]^{N-2} p_{r}^{2}e^{2t_{r}}+ N [\ldots]^{N-1}p_{r}e^{t_{r}}  $$

Letting *t*_*r*_=0 in this expression gives the second moment:

6 $$ \mathbb{E}(r^{2}) = N(N-1)p_{r}^{2} + Np_{r}  $$

Similarly,

7 $$ \mathbb{E}(l^{2}) = N(N-1)p_{l}^{2} + Np_{l}  $$

Using Eqs. 3, (6), (7), we obtain:

8 $$ \mathbb{V}\text{ar}(d) = Np_{r}(1-p_{r}) + Np_{l}(1-p_{l}) + 2 Np_{r}p_{l}  $$

In the case we are interested in, the walk is symmetric: *p*_*r*_=*p*_*l*_=*p*. Then:

9 $$ \begin{array}{lll} \mathbb{E}(d)&=&0\\ \mathbb{V}\text{ar}(d)&=&2Np \end{array}  $$

Each sequence generates, on average, *Np*_*i*_ insertions and *Np*_*d*_ deletions (*p*_*i*_ and *p*_*d*_ are respectively the probabilities of insertion and deletion). However, not all these indels produce gaps in the sequence alignment. For instance, 2 deletions at the same position in the sequences do not result in a gap. Also, an insertion in one sequence and a nearby deletion in the other sequence can result in a mismatch depending on the local sequence configuration (since it is better to incur a penalty for a mismatch than for 2 indels). Therefore, *p* is given by:

10 $$  p = 2 \times (p_{d} + p_{i} - p_{d} \times p_{d} - p_{i} \times p_{i} - O(p_{i},p_{d}))  $$

where *O*(*p*_*i*_,*p*_*d*_) is a term gathering the probabilities of cases similar to the last one above that are difficult to estimate beforehand. As shown in section “Results and discussion”, this term is small compared to the others in *p*.

Having estimated the standard deviation of *d*, *σ*_*d*_, we can choose *w*=3*σ*_*d*_. With this value of *w*, there is < 0.3% chance that the alignment path leaves the band. For the sake of illustration, *σ*_*d*_≃111 with reads of length *N*=30,000, using the PacBio error rates mentioned in the introduction (7% insertions, 4% deletions, i.e., *p*≃0.1035). The algorithm is thus $O\left (N^{\frac {3}{2}}\right)$ in time and space.

In practice, though, implementation details matter. As mentioned above, one can choose *w*=3*σ*_*d*_, leading to an algorithm whose execution time is proportional to 6*σ*_*d*_*N* (neglecting the above < 0.3% cases). Another possibility is, first, to choose *w*=*σ*_*d*_. On average, the alignment path remains in the band for 68% of the studied cases. For the remaining 32%, one restarts the computation with *w*=2*σ*_*d*_. For a further 28% of the cases, the alignment path remains in the band. For the last 4%, one restarts again the computation with *w*=3*σ*_*d*_. The total computation time is proportional to:

11 $$ \begin{aligned} {}2\sigma_{d}N &\times 0.68+ \left[2\sigma_{d}+4\sigma_{d}\right] N \times 0.28\\&\quad+ \left[2\sigma_{d}+4\sigma_{d}+6\sigma_{d}\right] N \times 0.04 = 3.52\sigma_{d} N \end{aligned}  $$

This scheme allows a gain of a factor ∼ 2 on the computing time.

### Is the score found by the banded DP algorithm optimal?

The usual answer found in text books and articles (e.g., [5]), valid for *global* alignments only, is the following.

Assuming that the length of the first sequence is *l*_1_ and the length of the second one is *l*_2_ (with *l*_1_>*l*_2_), a cell *M*[*i*,*j*] of the dynamic programming matrix is within the band if −*w*−(*l*_1_−*l*_2_)≤*i*−*j*≤+*w*. As shown on Fig. 1, the alignment path leaves the band if some of the cells on this path have *i* and *j* indexes such that *i*−*j*>*w* (dashed path) or *i*−*j*<−*w*−(*l*_1_−*l*_2_) (solid path). The best score obtained for such a path is given by:

12 $$ best_{out} = \left[2(w+1)-(l_{1}-l_{2})\right]g+ \left[l_{2}-(w+1)\right] m  $$

where *g* is the gap penalty and *m* is the match score. Indeed, this path generates *w*+1−(*l*_1_−*l*_2_) gaps in the horizontal sequence, *w*+1 in the vertical sequence and at most *l*_2_−(*w*+1) matches. Let *s*_*band*_ be the best score found by applying the dynamic programming algorithm in a band and *s*_*opt*_ be the best score obtained with the complete matrix. If the path does not leave the band, then *s*_*band*_=*s*_*opt*_. If *s*_*band*_≥*best*_*out*_ than *s*_*band*_ is optimal since it is larger than the best score of any possible alignment that travels outside of the band. Notice that the above formula provides an upper bound of *best*_*out*_, since it is assumed that all the aligned characters in the 2 sequences correspond to matches, excluding the possibility of mismatches.

**Fig. 2:**
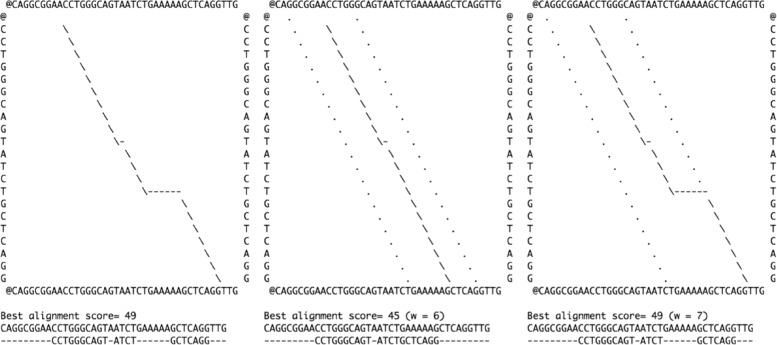
Semi-global alignments. Left: path of a semi-global alignment between two sequences using the full-matrix DP algorithm. The score function used is: match = +3, mismatch = -1, gap = -2. Middle: same as left but using the DP algorithm in a band. The band is not wide enough (w = 6) to fit the path. Right: same as middle, but this time the band can accommodate the path. Band limits are displayed with dotted lines

Using a semi-global alignment might often be more appropriate for the problem at hand. Figure 2 shows a semi-global alignment between 2 sequences, using the full DP algorithm on the left panel and using the DP algorithm in a band in the middle and right panels. In the middle panel, the band width, *w*=6, is not sufficient to fit the path. In the right panel, the band width has been increased to *w*=7 and the path remains in the band. Although this change is marginal, it has a dramatic impact on the alignment provided by the algorithm.

What criterion should one use to detect instances where the path leaves the band in semi-global alignments? In this case, the above global alignment criterion is not applicable since one does not know in advance where will be the ends of the path.

We tested 2 criteria. The first one is whether or not the path reaches the edge of the band (which is straightforward to check when one performs the backtracking procedure to find the path). The second criterion is related to the distribution of the number of matches in the alignments. The expected number of matches, *N*_*m*_, in alignments of sequences of length *N* and the corresponding variance are given by:

13 $$ \begin{array}{lll} \mathbb{E}(N_{m}) & = & Np_{u}\\ \mathbb{V}\text{ar}(N_{m}) & = & Np_{u}(1-p_{u}) \end{array}  $$

where *p*_*u*_=1−*p*_*m*_−*p*_*i*_−*p*_*d*_ is the probability of no modification of the nucleotides in the sequences. *p*_*u*_ is an upper bound of the true probability. Empirically, we checked that this provides a very good approximation of the mean of the number of matches. We consider that the path has left the band if the corresponding score is less than $\mathbb {E}(N_m) - 3\sigma _{N_{m}}$ (lower half of the 99% confidence interval).

With these 2 criteria, we propose the following procedure to decide whether the banded DP alignment has provided the optimal solution: i) we check whether the path reaches the band edge. If it is true, we restart the alignment with a larger value of *w*. If it is false, we further check whether the number of matches is outside the 99% confidence half interval. If it is true, we restart the alignment with a larger value of *w*, else we consider that the banded DP algorithm has found the optimal solution.

However, as shown in Fig. 2, the path can reach the edge of the band then move along it providing the optimal solution (right panel). Conversely, the alignment can provide a suboptimal solution although the path, apparently, does not reach the band edge (middle panel). To measure the magnitude of these effects, we performed a number of simulations as described in the next section.

## Results and discussion

### Test of the theoretical standard deviation

To check the validity of the assumption made in Eq. (10), we generated 8 sets of 1000 pairs of sequences having different lengths: 2000, 3000, 4000, 6000. Each set was generated using 3 different error rate sets: set1 = (0.85, 0.03, 0.075, 0.045), set2 = (0.93, 0.01, 0.04, 0.02), set3 = (0.89, 0.02, 0.06, 0.03) where the 4 figures within parentheses are respectively the probabilities of no modification (*p*_*u*_), a mismatch (*p*_*m*_), an insertion (*p*_*i*_) and a deletion (*p*_*d*_) of a nucleotide. We aligned all the pairs using the dynamic programming algorithm and we determined the maximal run of indels, in a particular direction, in the alignments, i.e., *d* above. For each set of 1000 aligned pairs of sequences we computed the mean and standard deviation of *d*. As awaited, the computed means are very close to zero. Simulation results for the standard deviations are shown in Table 1.

**Table 1:** Comparison of simulated and theoretical standard deviations

	error rate set 1		error rate set 2		error rate set 3	
Length	*σ*_*ex*_	*σ*_*th*_	*σ*_*ex*_	*σ*_*th*_	*σ*_*ex*_	*σ*_*th*_
2000	28.8 ± 0.3	30.0	20.2 ± 0.3	21.5	25.1 ± 0.3	26.1
3000	35.6 ± 0.5	36.7	25.2 ± 0.4	26.4	30.8 ± 0.3	32.0
4000	41.4 ± 0.5	42.4	28.8 ± 0.3	30.5	35.6 ± 0.5	37.0
6000	50.1 ± 0.7	51.9	35.5 ± 0.3	37.3	44.0 ± 0.5	45.3

As expected, theoretical standard deviations, *σ*_*th*_, calculated without the *O*(*p*_*i*_,*p*_*d*_) term are larger than the experimental ones (they are all outside the 99% confidence interval around the experimental mean value, *σ*_*ex*_). However, the difference is sufficiently small to be of no consequence for our purpose.

### Test of the two criteria for score optimality

To measure the pertinence of the two criteria described above, we used the results of the previous simulations. Table 2 shows the results for the 32,000 pairs of sequences generated with the 3rd error rate set (alignments were performed with *w*=*σ*_*d*_).

**Table 2:** Two-way table of banded DP alignments

	optimal score	suboptimal score
did not reach the band edge	68.4%	0.5%
reached the band edge	2.2%	28.9%

The proposed algorithm, would be optimal if the 2 off-diagonal elements contained 0% alignments. Here, 2.2% of the alignments provide the optimal solution and reach the band edge. It means that one will restart the alignment with a larger value of *w* although the result is correct. This wastes some time, but has no incidence on the correctness of the final alignment. On the contrary, when the path of the alignments does not reach the band and provides a suboptimal score (which is the case for 0.5% of the alignments) one will wrongly accept a suboptimal alignment.

Applying the 2nd criterion after the 1st criterion, the percentage of alignments that reach the band edge but provide an optimal score decreases from 2.2 to 1.8% and the percentage of alignments that do not reach the band edge but provide a suboptimal score drops from 0.5 to 0.2%, improving by 60% the latter problematic cases.

The procedure to determine whether the DP algorithm in a band finds the optimal score proposed in this note is thus not completely foolproof, but provides the correct answer in the vast majority of cases.

## Conclusion

The advent of third generation sequencing technologies that are characterized by long reads exhibiting high error rates, most notably for insertions and deletions, which are the most difficult errors to cope with, call for the development of new methods, better adapted to these features. Although the DP algorithm can hardly be defined as “new”, the problem at hand, aligning long reads that differ “only” by random sequencing errors, is ideally suited for using dynamic programming in a band.

In this note, we showed how to choose the band width in function of the reads’ error rates, resulting in a subquadratic time and space algorithm and proposed a procedure to determine whether the alignment path stays in the band, thus providing the optimal score. Therefore, the DP algorithm in a band is a good contender to align long reads between themselves, and can constitute a key component of methods for correcting long read sequencing errors and obtaining a consensus sequence (to be published).
